# Screening men in the Toledo district of southern Belize for elevated blood pressure and blood sugar

**DOI:** 10.1371/journal.pgph.0007380

**Published:** 2026-10-01

**Authors:** John Rovers, Matthew Nicasio

**Affiliations:** 1 Drake University, College of Pharmacy & Health Sciences, Des Moines, Iowa, United States of America; 2 Hillside Health Care International, Eldridgeville, Toledo District, Belize; National Center for Chronic and Noncommunicable Disease Control and Prevention, Chinese Center for Disease Control and Prevention, CHINA

## Abstract

Belize is a small country in Central America that has largely completed its epidemiological transition. Consequently, the Belizean burden of disease is increasingly composed of chronic illness, especially diabetes and hypertension. Recently, Belizean progress towards Sustainable Development Goal 3 (Good Health and Wellbeing) has slowed. Belizean men have 275 cardiac and 60 diabetes related deaths per 100,000 per year which are the third and fifth highest incidence in the Americas. Prevalence of raised fasting glucose is 8.5% in men (third highest in the Americas). Overall, 24% of Belizeans have an elevated blood pressure. In men, 20.3% have an elevated blood pressure. (12^th^ highest in the Americas). The objective of this study was to screen men in the Toledo district for both conditions. During visits to two local villages and at four health fairs held in the Punta Gorda town square, men were invited to have their blood pressure and blood sugar checked. Data was collected from 103 men. Systolic blood pressure ≥ 140mm/Hg was seen in 32 (31.1%) of men while diastolic blood pressure ≥ 90m mm/Hg was seen in 26 (25.2%) of men screened. Eight subjects (7.8%) had a random blood glucose ≥ 200mg/dl meriting further evaluation to rule out diabetes. Blood pressure and blood sugar were both statistically higher in men taking antihypertensive or antiglycemic medication. Hypertension and diabetes are common in men in the Toledo District and neither condition is well controlled in most men. Facilitators and barriers to control are largely related to social determinants of health. Men’s opinions about health and healthcare are often gender specific. Interventions to improve care in this setting should increase their focus on the social determinants of health and the gender specific beliefs and preferences men may have.

## Introduction

Belize is a small country in Central America. In 2022, its total midyear population was 441,471, of which males and females were nearly equivalent in numbers (220,739 male; 220,732 female) [1]. The Toledo district borders Guatemala and is the southernmost, and mostly rural district with 6,801 residents living in the capital, Punta Gorda, and 34,735 residents living in rural Toledo. Although Belize is considered an upper middle-income country by the World Bank with a Gross Domestic Product (GDP) per capita of $6,948, Toledo is the poorest district in the county with an estimated 60.3% of the population living in multi-dimensional poverty [2,3].

The Belizean burden of disease caused by noncommunicable disease, notably hypertension and diabetes continues to increase [4]. Given that the median age in Belize is only 26.4 years, as this young population continues to age, the consequence of increasing chronic disease is of growing concern. [5,6] Therefore, in collaboration with the Pan American Health Organization (PAHO), the Belizean Ministry of Health and Wellness’ Annual Report for 2020 has as its third priority, “Health and wellness promoted throughout the life course using an integrated primary healthcare approach to reduce noncommunicable diseases (NCD) and their risk factors, communicable diseases, mental health disorders, urban violence, and injuries.” [7]

Reliable health data for Belize are readily available but relatively little is disaggregated by sex or by district. As the literature below shows, some data is disaggregated by sex, but not by district, while other data is disaggregated by district, but not by sex. Although national level data can provide perspective on the health status of a country, health indicators for a community may be different than those gathered at the national level [8]. Morey and colleagues evaluated ethnic and gender disparities in Belize for adult premature mortality in the early 2000’s [9]. They found the overall risk of premature death for men aged 15–59 was 22.7%. Mayan men, the most common ethnic group in the Toledo district, had the lowest risk of premature death at 10.2%. More recently, Dekker and colleagues performed a mixed-methods assessment of Type II diabetes and hypertension care in the Toledo district. Although specific to the Toledo region, their results were not disaggregated by sex. Overall, they found the prevalence of both hypertension and Type II diabetes was 12%. [10].

The Sustainable Development Goals (SDGs) are a list of 17 goals and 169 targets for human development to be reached by 2030. SDG 3 is the goal related to health [11]. By 2018, Belize had met 68% of all 17 SDGs. However, by 2023, progress had decreased to 65% of goals met. Belize’s progress to date on SDG 3 (Good Health and Wellbeing) has been modest with many indicators stagnating or progress increasing at less than 50% of the desired rate [12].

Belize has largely completed its epidemiological transition and the bulk of the burden of disease is now chronic disease or injury [13,14] According to PAHO, deaths from non-communicable diseases (NCDs) in Belize are the third highest in the Americas with 750 men per 100,000 dying annually from an NCD. [15] Men aged 30–70 have a 23% chance of dying from an NCD and 47.5% of NCD deaths are in people under aged 70. Belizean men have 275 cardiac and 60 diabetes related deaths per 100,000 per year which are the third and fifth highest incidence in the Americas. Prevalence of raised fasting glucose is 8.5% in men (third highest in the Americas). Overall, 24% of Belizeans have an elevated blood pressure. In men, 20.3% have an elevated blood pressure. (12^th^ highest in the Americas). Leading causes of death are cardiovascular disease (13.6%), malignant neoplasms (10.0%), unintentional injury (6.6%), and diabetes mellitus (6.2%). [1]

According to the Global Burden of Disease data, diabetes in Belize accounts for 5.64% of total Disability Adjusted Life Years (DALYs) (4.49% in men) and is increasing at a rate of 2.51%. Hypertensive heart disease accounts for 1.31% of total DALYs (1.23% in men) and is also increasing by 2.51% annually. [16]

### Objective

Given the high prevalence of hypertension and diabetes mellitus in Belizean men, the objective of this study was to screen men in the Toledo district for both conditions. Previous screenings for diabetes and hypertension were not disaggregated for men or did not take place in the Toledo district. Since hypertension and elevated plasma glucose are common co-morbidities, if screening for one condition it is well advised to include screening for the other [17,18]. Results specific to men’s health in Toledo will allow for more accurately targeted interventions to educate Toledo men on their health and manage their conditions better.

## Methods

### Ethics statement

This study was approved by the Drake University Institutional Review Board (IRB Proposal #: 2022–23055) The Director of Health Services from the Belizean Ministry of Health was contacted on multiple occasions to request local permission, as were local medical employees of the Ministry but no response was ever received. Five community members served as an advisory committee to ensure the work was culturally and socially appropriate. This study adhered to the requirements of the Declaration of Helsinki.

### Study setting

This study was carried out in collaboration with Hillside Health Care International which operates Hillside Clinic [19]. Hillside Clinic is a non-governmental organization (NGO) located near Punta Gorda, which is the largest town in the Toledo District. Hillside Clinic provides Toledo residents with free, year-round services in home health care, public health, rehabilitation, medical care, mobile clinics, community outreach, and pharmacy.

In 2024, Hillside Clinic provided care to 3935 patients in the outpatient clinic, 1304 patients during mobile clinics, 1499 home health nursing visits, and 1351 rehabilitation services. Punta Gorda residents represented 9–14% of outpatient clinic visits each quarter. Men received 21% of outpatient clinic services.

As shown on the map in Fig 1, Hillside Clinic and Punta Gorda town are both on paved roads and readily accessible by regular bus service from outlying villages. Hillside Clinic provides scheduled mobile visits to several villages, all of which are on dirt roads. In addition, there are several polyclinics located throughout Toledo District as well as a hospital in Punta Gorda. Punta Gorda has a centrally located town square that affords easy access to men who come into town on Saturdays to transact business or shop for supplies.

**Fig 1:**
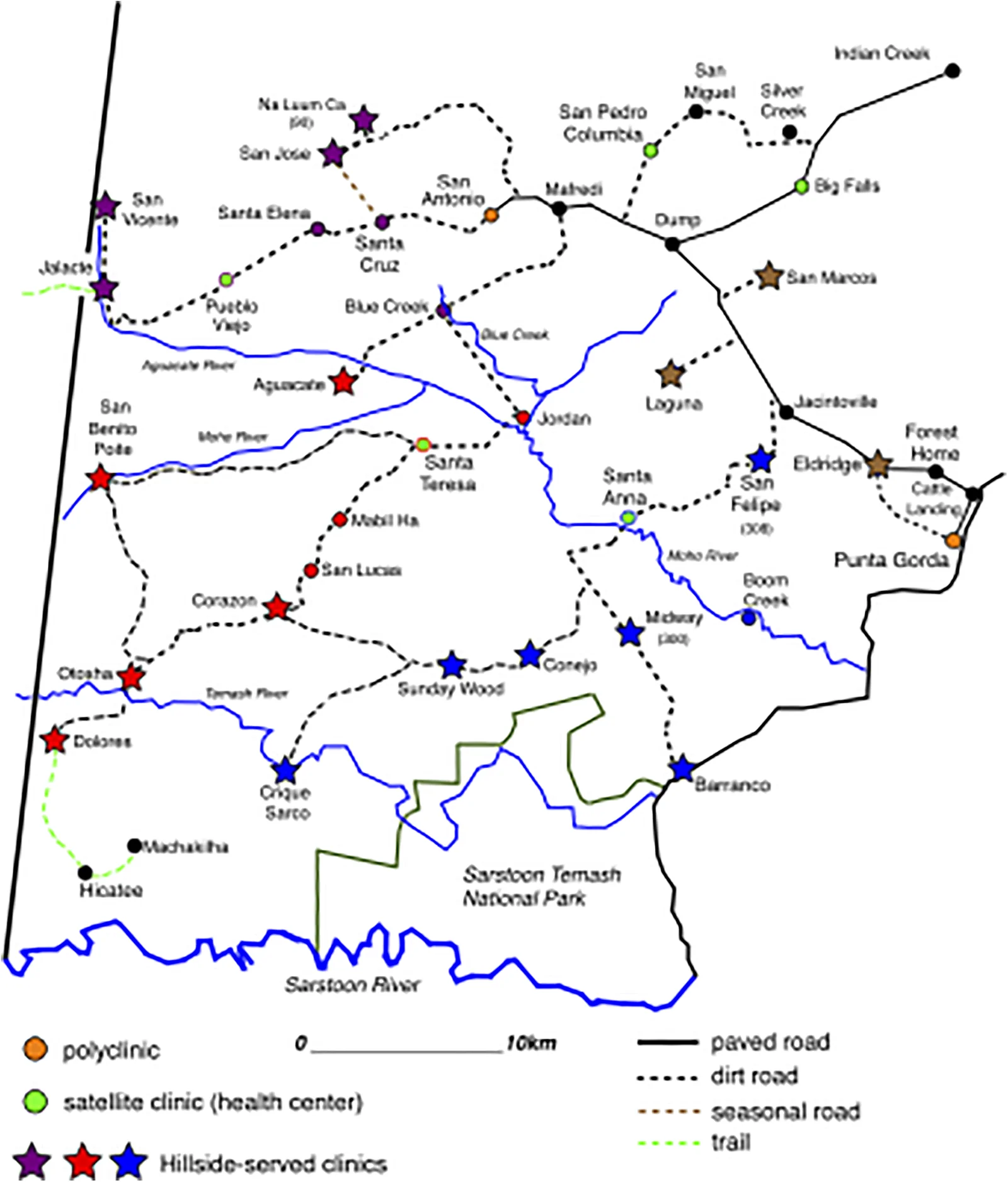
Map of Toledo District and towns served by Hillside.

Informal information provided by Hillside staff indicates that relatively few men seek care, either in clinic or during a mobile visit to a village. This is thought to be a function that most men in Toledo are subsistence farmers and unwilling or unable to give up a day’s work in the field to attend to health problems. This may also be a function of men generally being reluctant to seek out health care services when ill [20–22].

### Data collection

Data was collected from a convenience sample of men who were available and willing to be screened between August 5, 2023, and February 26, 2024, in two villages (Big Falls and Silver Creek) and four health fairs offered monthly in the town square in Punta Gorda. Community Health Workers (CHWs) in the villages promoted the events to the men in their village. During the health fairs, men passing through were invited to participate, have their blood pressure and blood sugar checked, and receive education on hypertension and diabetes.

Inclusion criteria included: male sex, age ≥ 18 years, sufficient English ability to converse and understand study procedures and the consent form, no use of anticoagulants that may affect finger stick blood draw, no active infection or fever ≥ 100.5° F.

The following data were collected from each participant: age, informed consent provided, systolic and diastolic blood pressure, random blood sugar, history of hypertension and/or diabetes, use of antihypertensive and/or antidiabetic medication, if referral was made for medical follow-up by a physician, and if patient education on blood pressure and/or blood sugar was provided. Blood pressure was measured using a McKesson Automatic Digital Blood Pressure Monitor while blood sugar was measured using an Embrace Pro Blood Glucose Meter and appropriate test strips. All tests were done with the subject in a sitting position. Testing was carried out by Hillside medical and nursing staff as well as senior level clinical students from various health disciplines.

All subjects provided written, informed consent prior to any testing being undertaken. (Drake IRB Proposal # 2022–23055).

Statistical testing was carried out by one-way Analysis of Variance (ANOVA) with Bonferroni correction or Kruskal-Wallace test with Dunn’s test and Bonferroni correction. Significance was set at p ≤ 0.05.

## Results

Useable data was collected from 103 men. Demographic information for all subjects is shown in Table 1. As shown, most were middle aged.

**Table 1:** Demographics.

Subject Demographics (n103)	Age
Mean Age (Years)	50.5
Median Age (Years)	52.0
Mode Age (Years)	41
Minimum Age (Years)	19
Maximum Age (Years)	74
Age Distribution	(n)
18-24	4
25-34	5
35-44	28
45-54	22
55-64	26
≥ 65	18

Table 2 includes the total number of subjects with systolic and blood pressures greater than 140mmg/Hg or 130 mm/Hg or diastolic blood pressures greater than 90mmg/Hg or 80mmg/Hg. Table 2 also includes the number of subjects with a blood glucose greater than 200mg/dl or between 100 and 126mg/dl. It also shows the number of subjects in whom the blood glucose measurement was fasting, random, or unknown.

**Table 2:** Aggregate screening results (SBP – Systolic blood pressure, DBP – Diastolic blood pressure, BG – Blood glucose).

Blood Pressure (mm/Hg) & Blood Glucose (mg/dl)	n	%
SBP ≥ 140	32	31.1
DBP ≥ 90	26	25.2
SBP ≥ 130	44	42.7
DBP ≥ 80	44	42.7
Random BG	79	76.7
Fasting BG	2	1.9
Unknown BG	22	21.4
Random BG ≥ 200	8	7.8
Fasting BG 100–126	1	1

As shown in Table 2, 32 (31.1%) and 26 (25.52%) of subjects respectively had a systolic or diastolic blood pressure measurement consistent with hypertension while 44 (42.7%) of subjects had a systolic or diastolic measurement consistent with a high normal blood pressure. Eight subjects (7.8%) had a random blood glucose ≥ 200mg/dl meriting further evaluation to rule out diabetes.

Table 3 disaggregates most of the data from Table 2 by age.

**Table 3:** Screening results disaggregated by age (SBP – Systolic blood pressure, DBP – Diastolic blood pressure, BG – Blood glucose).

Age (yrs)	n	Mean SBP (mm/Hg)	Range SBP (mm/Hg)	Mean DBP (mm/Hg)	Range DBP (mm/Hg)	Mean BG (mg/dl)	Range BG (mg/dl)
18-24	4	144	115-175	80	63-93	140	125-158
25-34	5	120	103-145	79	66-84	125	104-145
35-44	28	127	90-162	78	57-100	138	96-306
45-54	22	138	103-185	83	61-118	151	93-377
55-64	26	126	90-175	77	83-104	156	87-568
≥ 65	18	129	111-166	83	59-108	127	83-239

As shown in Fig 2, both mean systolic and diastolic pressures showed a possible trend upwards with age as did mean blood glucose. One-way ANOVA was not significant for SBP (p = 0.562) or DBP (p = 0.607) and approached significance for BG (p = 0.0503). However, after applying the Bonferroni correction for multiple comparisons, no significant pairwise differences in BG were seen.

**Fig 2:**
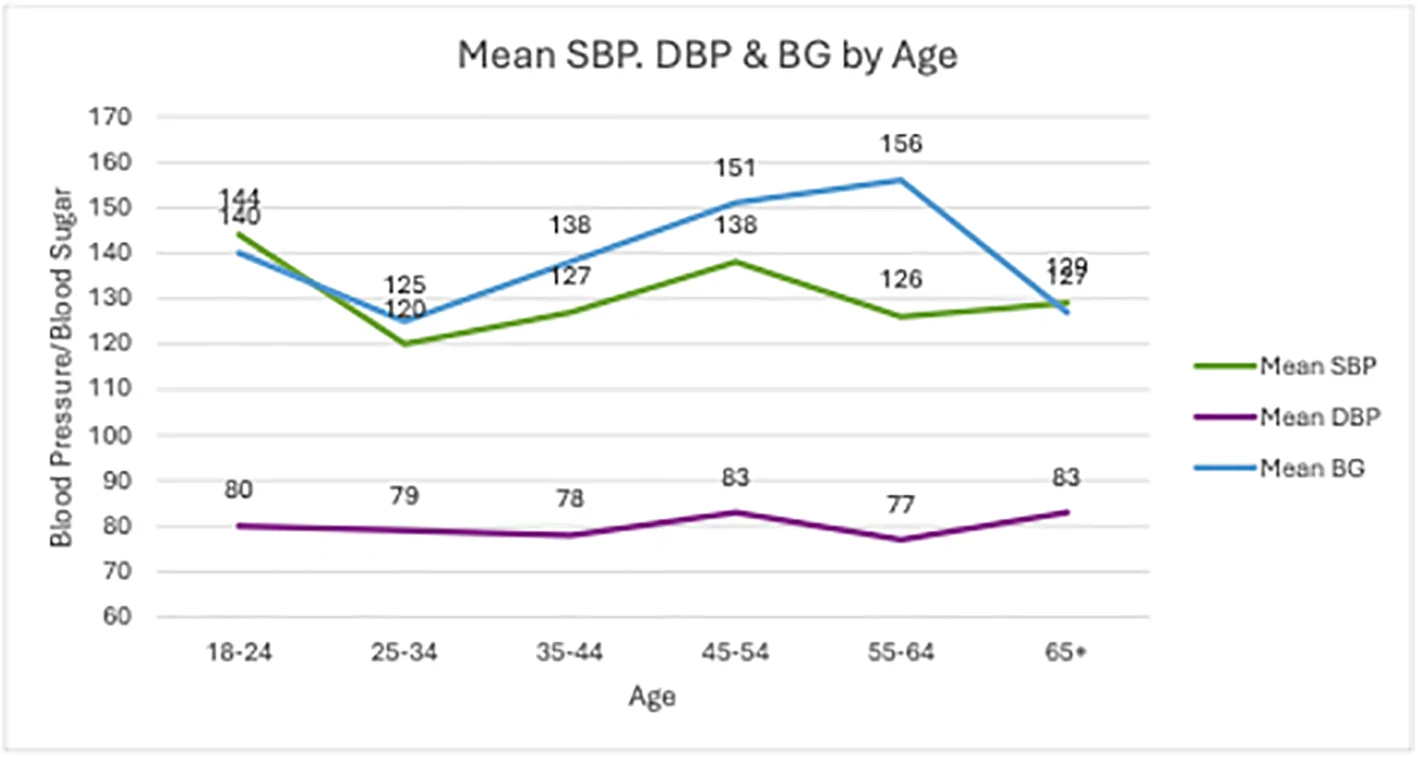
Mean screening results disaggregated by age (SBP – Systolic blood pressure, DBP – Diastolic blood pressure, BG – Blood glucose).

Table 4 shows the mean results for SBP, DBP, and random BG in all men depending on whether they were taking medication for one or more conditions or not taking any medication. The Kruskal-Wallis test confirmed significant group differences for all three medication related variables. (SBP p = 0.018206, DBP p = 0.045402, BG p = 0.010733).

**Table 4:** Mean screening results disaggregated by medication use (SBP – Systolic blood pressure, DBP – Diastolic blood pressure, BG – Blood glucose, SD – Standard deviation).

	n	Mean SBP + /- SD	Mean DBP + /- SD	Mean Random BG + /- SD
Taking medication	10	147 + /- 19	90 + /-12	204 + /- 45
Not taking medication	37	129 + /-20	80 + /-13	135 + /- 78
Unknown medication use	46	125 + /-20	77 + /-14	137 + /-75

The Dunn’s post-hoc test with Bonferroni correction identified several significant pair-wise comparisons. The results are shown in Table 5. Mean SBP, DBP, and BG were significantly higher in all men taking medication compared to unknown medication use. Men taking medication also had significantly higher BG than men not taking medication, while SBP approached significance as well.

**Table 5:** Statistical differences disaggregated by medication use (SBP – Systolic blood pressure, DBP – Diastolic blood pressure, BG – Blood glucose) * = significant difference.

Medication use comparisons	SBP (p value)	DBP (p value)	BG (p value)
Not taking medication vs Taking medication	0.058899	0.193283	0.045242 *
Not taking medication vs Unknown medication use	1.0	0.889097	0.976412
Taking medication vs Unknown medication use	0.01394 *	0.040941 *	0.00783 *

All subjects were provided with general education about diabetes and hypertension while 32 (31.1%) were advised to see a physician or attend a clinic at Hillside for follow-up.

## Discussion

As the prevalence of diabetes and hypertension continues to increase in underserved areas, community-based interventions including screening programs have been suggested as a promising activity to bridge gaps in care [23].

Our results suggest that hypertension and diabetes mellitus are common conditions in men in the Toledo district and that both conditions may not always be well controlled. We found that 32 (31.1%) and 26 (25.52%) of subjects respectively had a systolic or diastolic blood pressure measurement consistent with hypertension while 44 (42.7%) of subjects had a systolic or diastolic measurement consistent with a high normal blood pressure. Eight subjects (7.8%) had a random blood glucose ≥ 200mg/dl meriting further evaluation to rule out diabetes. These data are consistent with previous research carried out in Belize and neighboring regions.

In a study in the Toledo district of both men and women, Dekker and colleagues found the prevalence of both hypertension and diabetes was 12% [10]. Of their subjects, 51% of hypertensive patients were at blood pressure goal and 26% of diabetic patients were at their glycemic goal. In an older study carried out in a neighboring district in Belize, Chung and colleagues found 11/38 (28.9%) of men had a history of hypertension, and 20/38 (52.6%) were newly diagnosed during screening or had uncontrolled hypertension. Only 1/11 (9.1%) of treated hypertensive men had adequately controlled blood pressure [24].

In a multinational study of diabetes, Barcelo and colleagues found the prevalence of diabetes in Belize was 12.5% [25]. The prevalence of diagnosed and undiagnosed diabetes in Belizean men was 4.7% and 3.6% respectively.

In a chart review study in neighboring Guatemala, Chary and colleagues found that 12.8% and 17.9% of subjects had a SBP and DBP ≥ 140mmg/Hg and ≥ 90mm/Hg respectively. The average glycosylated hemoglobin (HbA 1c) was 8.9 + /- 2.1% [26].

The present study and related literature suggest both hypertension and diabetes are common in Toledo and neighboring regions and that neither condition is particularly well managed. This is despite Belize having an integrated, country wide health information system that embeds care protocols for a variety of conditions including hypertension, although not diabetes [27]. In the Belizean public system, as well as Hillside Clinic, health care is free at the point of care and prescription drugs are provided at no cost. The Toledo district has two polyclinics, a small hospital, and a CHW in all villages. Thus, health care is readily available and is free. This then begs the question as to why diabetes and hypertension remain common and not always well controlled.

A systematic review of barriers and facilitators to care for diabetes in Latin America and the Caribbean region uncovered 13 domains that can either facilitate or form a barrier to care [28]. The most common domain identified was “environmental context and resources” in which three themes emerged: health system context, patient context, and environmental context. System context included availability of resources, time, communications, and universal health coverage. Patient context included the financial situation, need for stability to attend scheduled care visits, employment status, and the need for dietary and exercise changes. Environmental context included green space suitable for exercise, and availability of suitable food. Barriers in the social domain included lack of support from family and friends, social pressure to disrupt diet at social functions, stigma, and the need for peer support. Other domains identified include a patient’s social or professional role, their knowledge about diabetes, their ability to regulate their activities to manage their condition, the emotional burden of the disease, their beliefs about the consequences of diabetes and their level of optimism, their sense of self-efficacy, and their intention to change habits.

In a qualitative study of the voices of Belizeans with Type II diabetes, Allen and colleagues identified several barriers to care [29]. Some respondents noted that not all Belizeans had basic coverage under the National Health Insurance scheme, without which care was unaffordable. They also voiced a need for greater and better disseminated public education about diabetes.

In a systematic review of pathways for hypertension care in low- and middle-income countries, Brathwaite and colleagues noted a problem with how hypertension is managed in such countries [30]. They note that most research describes how to get patients into the healthcare system to be treated but does not evaluate what happens after such individuals formally enter the system. In many studies (including this one) patients are successfully screened for hypertension, but follow-up is inadequate. Once therapy has started for those who have entered the system, financial status, lack of education, co-morbidities, the lack of symptoms in hypertension, inadequate staffing, equipment and medication, patient knowledge and beliefs about hypertension, the need for trade-offs between self-care and family and work responsibilities, and social relationships and traditions all affect the success of patient management. The long-term management of those undergoing treatment is likewise affected by demographics and socio-economic status, the patient’s health status and comorbidities, numerous health system factors, and individual knowledge and beliefs.

In a scoping review of interventions targeting hypertension and diabetes in low- and middle-income countries, Correia and colleagues note the literature on the topic is heterogeneous, complex, and usually does not provide sufficient context to allow for implementation of care processes or study replication. [31] This makes improving patient care processes challenging.

Given the high prevalence of hypertension and diabetes in Belize and the literature describing facilitators and barriers to care, it would be helpful to identify interventions that may improve patient care. Many of the barriers and facilitators to care cited above could be categorized into a single factor – social determinants of health (SDOH) [32]. Although comorbidities, patient knowledge and attitudes towards illness, and willingness to change dietary and exercise habits can serve as a barrier or facilitator to care, the literature makes it clear that SDOH probably play a bigger role. Once a patient has been screened into the healthcare system, someone’s available time, education, employment status, family and work responsibilities, social relationships, and cultural practices and traditions can all affect their clinical outcome. Similarly, health system factors including access to suitable providers, distance to a clinic or hospital, access to medical supplies and medications, and health insurance status are often barriers to effective care. It is apparent that although patients must receive competent clinical care to effectively manage their hypertension and diabetes in low resource settings, scalable and replicable interventions play an equally, if not more important role, in managing chronic disease.

Although it is not specific to Belize or Central America, in addition to these SDOH factors, unique health beliefs and preferences in men need to be considered since health beliefs are often gendered [21,33,34]. A study in Norwegian men found that subjects conceptualized health in four different domains [35]. They saw health as: (1) adequate functionality and the ability to live life as desired; (2) absence of limiting illness, injury, and pain; (3) good social relations and family, and a sense of belonging; (4) an acceptable body shape and weight. Since hypertension is asymptomatic, and does not impair the ability to work, these health beliefs must be specifically addressed to prevent long-term sequelae of the disease. A group of African American men stated their health information needs as having community norms about male roles addressed [36]. A sense of trust in the healthcare system was also important. Shepherd describes a “roadshow” focusing specifically on men’s health and notes that the interventions need to be gender specific, pragmatic, and flexible [20]. Since hypertension and diabetes are often managed by improvements in diet and exercise, a study in Danish men showed that subjects saw health as a broader construct than making behavioral changes [37]. The same authors also noted that men with health concerns want to be talked to, not talked at and that appropriate recognition of gender and masculinity was desired [38]. Being condescended to and disrespected was common in these men’s experience and resented by them. One stated, “The second I became a patient, they thought I was retarded.” Whitely and colleagues recommend that men’s health programs demonstrate concern, build rapport, engage in productive dialog, advocate for necessary policy changes, and be culturally competent. [39]

### Limitations

There are several limitations of this study. Diabetes was assessed using a random, fingerstick blood glucose. As such the actual prevalence of diabetes and sugar control may be different than what our results suggest. A fasting blood glucose or an HbA 1c level would have been preferable, but lack of the necessary testing equipment and the screening environment made this unfeasible. Since men choosing to be screened were not generally patients of Hillside Clinic, adequate follow-up for those with elevated blood pressures or blood sugars was not possible. Instead, all men were provided with appropriate education and if necessary, referred to the clinic or a local physician for follow-up care.

## Conclusion

Blood pressure and blood sugar in men in the Toledo District of Southern Belize are frequently not well controlled. Numerous barriers and facilitators to care can be identified in the literature. Many such barriers or facilitators are grounded in SDOH. Toledo has a CHW in every village, there are several polyclinics throughout the district, and there is a hospital in Punta Gorda. Healthcare and medication are provided free of charge at Hillside and in government clinics. This suggests that poor control of blood pressure and blood sugar is unlikely to be a result of unaffordability, poor access to healthcare services, or medication. The results of this study will be shared with Hillside Clinic, local health officials, CHWs, and government agencies to design interventions to improve diabetes and hypertension care. The literature suggests these interventions should address relevant SDOH with special focus on the gender specific requirements men are likely to have. Such interventions may also be improved by basing them on data that reflects the local population as closely as possible, which may require data disaggregated from international or national resources.
