# Supplementary material for: Screening men in the Toledo district of southern Belize for elevated blood pressure and blood sugar
Source: PLOS Glob Public Health. 2026 Oct 1;6(10):e0007380. doi: 10.1371/journal.pgph.0007380 (PMC13630257; doi:10.1371/journal.pgph.0007380)
Supplement: Supplementary file 1 [file pgph.0007380.s001.docx]

**Reviewer Recommendation and Comments for Manuscript Number PGPH-D-25-02214**

Many congratulations to the authors on the completion of the project and on putting out this report.

However, I have a few comments.

| s/n | Comments | Recommendations |
| --- | --- | --- |
| 1 | The introduction is largely well written, but has conflicting statements.  Line 85 suggests that disaggregated data are not available, hence the need for the study, but the paragraph starting from line 85 says otherwise. So one is unsure what the justification for the study really is. | Review the introduction to clearly show the background, problem statement, and justification. |
| 2 | The objective of the study does not make it seem like research. An objective just to screen men for certain conditions is too simplistic. | Consider reviewing the objective to look less than just a medical outreach. What do you hope to achieve with the screening? |
| 3 | Methods are not clear on the sampling, sampling method, and sample size. | It might be good to include all of these |
| 4 | Ethical Approval was unclear. If the approval from the university covers the area, there may not be a need to mention the one you did not get. Unless it does not, in which case, you do not have ethical approval for this. | Consider reviewing this. |

In addition, the results and discussion would follow the objective (s), and in this case, it was unclear to follow. Based on this, the author could not have concluded as they did.
