# Supplementary material for: Screening men in the Toledo district of southern Belize for elevated blood pressure and blood sugar
Source: PLOS Glob Public Health. 2026 Oct 1;6(10):e0007380. doi: 10.1371/journal.pgph.0007380 (PMC13630257; doi:10.1371/journal.pgph.0007380)
Supplement: Supplementary file 2 [file pgph.0007380.s003.docx]

# Responses to Editor and Reviewers

## Editor’s Changes

1. Please include a complete copy of PLOS’ questionnaire on inclusivity in global research in your revised manuscript.
   a. A completed questionnaire on inclusivity in global research has been uploaded with the manuscript.
2. Please amend detailed Financial Disclosure Document
   a. This has been amended as requested. Sources of funding, author initials, role of funders, salaries from funders have all been amended as necessary or relevant.
3. Competing interest statement
   a. Completed as requested.
4. Data availability
   a. All data are available from Figshare using doi10.6084/m9.figshare.30659099
5. Figure files
   a. One figure (map) has been added to the submission as a .tif file.
6. Cite specific citations
   a. Not applicable

## Reviewer #1

1. Short title
   a. The short title has been amended as the reviewer suggested. It now reads:
      i. *Short Title: Hypertension and Diabetes Screening Among Toledo Men, Belize*
2. The study setting mainly described the facility where the data was going to be collected; however, it will be important to mention other important factors to consider….
   a. This has been amended as the reviewer suggests on page 7, lines 139-151. In addition, a map has been included to show the location of local community, health outposts, and roads. This map was created by Hillside Clinic and is reproduced with permission. It now reads:
      i. *In 2024, Hillside Clinic provided care to 3935 patients in the outpatient clinic, 1304 patients during mobile clinics, 1499 home health nursing visits, and 1351 rehabilitation services. Punta Gorda residents represented 9-14% of outpatient clinic visits each quarter. Men received 21% of outpatient clinic services.*

*As shown on the map in Figure 1, Hillside Clinic and Punta Gorda town	are both on paved roads and readily accessible by regular bus service	from outlying villages. Hillside Clinic provides scheduled mobile visits to	several villages, all of which are on dirt roads. In addition, there are	several polyclinics located throughout Toledo District as well as a	hospital in Punta Gorda. Punta Gorda has a centrally located	town square that affords easy access to men who come into	on Saturdays to transact business or shop for supplies.*

*Figure 1 Map approximately here.*

3. Relationship between blood pressure and blood glucose.
   a. This has been addressed on Page 6, Lines 126-128. In addition, 2 new references (#17 and #18) have been added. The section now reads:
      i. *Since hypertension and elevated plasma glucose are common co-morbidities, if screening for one condition is well advised to include screening for the other. (17,18) Results specific to men’s health in Toledo will allow for more accurately targeted interventions to educate Toledo men on their health and manage their conditions better.*
4. Additional variables could include occupation, marital status, existing diagnoses etc.
   a. The reviewer’s point is well taken, but this data was not collected and cannot be included. Table 4 does show that 10 of 103 subjects were taking medication for either hypertension or diabetes, but no further information is available.

## Reviewer #2

1. Is available data disaggregated or not?
   a. The reviewer’s confusion is well taken. This has been clarified on page 5, lines 87-88. The amended wording now reads:
      i. *As the literature below shows, some data is disaggregated by sex, but not by district, while other data is disaggregated by district, but not by sex.*
2. Objective is unclear and does not match conclusions.
   a. The reviewer is correct. This has been addressed on page 6, lines 127-129. The amended text now reads:
      i. *Results specific to men’s health in Toledo will allow for more accurately targeted interventions to educate Toledo men on their health and manage their conditions better.*
   b. The reviewer’s concern is also addressed in an amended conclusion on page 19, lines 392-398. The amended text now reads:
      i. *The results of this study will be shared with Hillside Clinic, local health officials, CHWs, and government agencies to design interventions to improve diabetes and hypertension care. The literature suggests these interventions should address relevant SDOH with special focus on the gender specific requirements men are likely to have. Such interventions may also be improved by basing them on data that reflects the local population as closely as possible, which may require data disaggregated for international or national resources.*
   c. We would also point out that on page 4, lines 88-90, Reference 8 notes that community level health indicators may be different from national data. Thus, appropriate and effective health interventions should reflect the local population as closely as possible. This will require disaggregated data.
   d. It may also be of interest to note that this project is a follow-up study to a previous project currently undergoing peer review elsewhere. In that study, we were able to identify several location specific SDOHs that can be applied to the results of the current study to create socially and culturally appropriate health education interventions for Toledo men. E.g. providing health education to men in Toledo requires that services be offered on Sundays. At any other time, they are either in the fields or elsewhere. Sunday is the only day one is likely to find them at home.
3. Sampling methods are unclear.
   a. This has been addressed on page 8, lines 159-160. The amended text now reads:
      i. *Data was collected from a convenience sample of men who were available and willing to be screened.*
   b. The number of men who were approached for screening and who declined is not available. However, the results on page 9, line 194 state useable data was collected from 103 men.
4. Ethical approval.
   a. The reviewer’s concerns are very well taken, and this has been a source of uncertainty for other projects carried out by Hillside Clinic. Hillside works closely with the Ministry of Health and Wellness and meets with officials regularly. Despite this, Hillside staff have struggled to determine the appropriate process to seek Ministry ethical approval for research projects. We share the reviewer’s concerns and acknowledge that research done in low resource, high needs settings is generally required to seek local official approval.

As noted in the manuscript, the project was approved by the Drake University Institutional Review Board (IRB Proposal # 2022-23055). The relevant approval letter has been submitted with this manuscript. In addition, we would point out that one of the investigators (Mr. Nicasio) was a member of the local community and able to ensure the work was carried out consistent with local ethical expectations. Also, as best practices suggest, he was a co-principal investigator and an author on the final manuscript. We also had an advisory committee of local residents who reviewed all work to ensure adherence to ethical standards. We sought advice from local medical officers employed by the Ministry without receiving a response. We note that the work adhered to the requirements of the Declaration of Helsinki. As best practices require, the results of the study will be made available to Belizean stakeholders allowing them to reap any benefits from the work. Essentially, we made every good faith effort possible to adhere to all standard ethical requirements. If this is not acceptable to the journal or to the reviewer, then so be it. But we tried to the best of our ability to comply with best practices in global health research. The ethical approval statement on page 9 lines 189-191 now reads:

i. *…….. as were local medical employees of the Ministry but no response was ever received. Five community members served as an advisory committee to ensure the work was culturally and socially appropriate.*

5. Results and discussion do not follow objectives.
   a. This has been addressed in Point 2 above.

# Reviewer #3

1. Methods section not clear. Clarity of recruitment process.
   a. This has been addressed as noted above under Reviewer #2, point 3.
2. The conclusions address the objectives of the study.
   a. This has been addressed as noted above under Reviewer #2, point 2.
