# Supplementary material for: Screening men in the Toledo district of southern Belize for elevated blood pressure and blood sugar
Source: PLOS Glob Public Health. 2026 Oct 1;6(10):e0007380. doi: 10.1371/journal.pgph.0007380 (PMC13630257; doi:10.1371/journal.pgph.0007380)
Supplement: Peer review history

# Peer review: Decision Letter 0

**DOI:** 10.1371/journal.pgph.0007380.r001

**Authors:** Pengpeng Ye

24 Sep 2025

PGPH-D-25-02214

Screening Men in the Toledo District of Southern Belize for Elevated Blood Pressure and Blood Sugar

PLOS Global Public Health

Dear Dr. Rovers,

Thank you for submitting your manuscript to PLOS Global Public Health. After careful consideration, we feel that it has merit but does not fully meet PLOS Global Public Health’s publication criteria as it currently stands. Therefore, we invite you to submit a revised version of the manuscript that addresses the points raised during the review process.

Please submit your revised manuscript by Nov 08 2025 11:59PM. If you will need more time than this to complete your revisions, please reply to this message or contact the journal office at globalpubhealth@plos.org. When you're ready to submit your revision, log on to https://www.editorialmanager.com/pgph/ and select the 'Submissions Needing Revision' folder to locate your manuscript file.

Please include the following items when submitting your revised manuscript:

- A rebuttal letter that responds to each point raised by the editor and reviewer(s). You should upload this letter as a separate file labeled 'Response to Reviewers'.
- A marked-up copy of your manuscript that highlights changes made to the original version. You should upload this as a separate file labeled 'Revised Manuscript with Track Changes'.
- An unmarked version of your revised paper without tracked changes. You should upload this as a separate file labeled 'Manuscript'.

Guidelines for resubmitting your figure files are available below the reviewer comments at the end of this letter.

We look forward to receiving your revised manuscript.

Kind regards,

Pengpeng Ye

Academic Editor

PLOS Global Public Health

Journal Requirements:

1. Please include a complete copy of PLOS’ questionnaire on inclusivity in global research in your revised manuscript. Our policy for research in this area aims to improve transparency in the reporting of research performed outside of researchers’ own country or community. The policy applies to researchers who have travelled to a different country to conduct research, research with Indigenous populations or their lands, and research on cultural artefacts. The questionnaire can also be requested at the journal’s discretion for any other submissions, even if these conditions are not met.  Please find more information on the policy and a link to download a blank copy of the questionnaire here: https://journals.plos.org/plosone/s/best-practices-in-research-reporting. Please upload a completed version of your questionnaire as Supporting Information when you resubmit your manuscript.

2. Please amend your detailed Financial Disclosure statement. This is published with the article. It must therefore be completed in full sentences and contain the exact wording you wish to be published.

1. Please clarify all sources of funding (financial or material support) for your study. List the grants (with grant number) or organizations (with url) that supported your study, including funding received from your institution.

2. State the initials, alongside each funding source, of each author to receive each grant.

3. State what role the funders took in the study. If the funders had no role in your study, please state: “The funders had no role in study design, data collection and analysis, decision to publish, or preparation of the manuscript.”

4. If any authors received a salary from any of your funders, please state which authors and which funders.

If you did not receive any funding for this study, please simply state: “The authors received no specific funding for this work.”

3. Please send a completed 'Competing Interests' statement, including any COIs declared by your co-authors. If you have no competing interests to declare, please state "The authors have declared that no competing interests exist". Otherwise please declare all competing interests beginning with the statement "I have read the journal's policy and the authors of this manuscript have the following competing interests:"

4. When completing the data availability statement of the submission form, you indicated that you will make your data available on acceptance. We strongly recommend all authors decide on a data sharing plan before acceptance, as the process can be lengthy and hold up publication timelines. Please note that, though access restrictions are acceptable now, your entire data will need to be made freely accessible if your manuscript is accepted for publication. This policy applies to all data except where public deposition would breach compliance with the protocol approved by your research ethics board. If you are unable to adhere to our open data policy, please kindly revise your statement to explain your reasoning and we will seek the editor's input on an exemption. Please be assured that, once you have provided your new statement, the assessment of your exemption will not hold up the peer review process.

5. Please provide separate figure files in .tif or .eps format.

For more information about figure files please see our guidelines:

https://journals.plos.org/globalpublichealth/s/figures

https://journals.plos.org/globalpublichealth/s/figures#loc-file-requirements

6. If the reviewer comments include a recommendation to cite specific previously published works, please review and evaluate these publications to determine whether they are relevant and should be cited. There is no requirement to cite these works unless the editor has indicated otherwise.

[Note: HTML markup is below. Please do not edit.]

Reviewers' comments:

Reviewer's Responses to Questions

**Comments to the Author**

1. Does this manuscript meet PLOS Global Public Health’s publication criteria?

Reviewer #1: Yes

Reviewer #2: No

Reviewer #3: Yes

**********

2. Has the statistical analysis been performed appropriately and rigorously?

Reviewer #1: No

Reviewer #2: I don't know

Reviewer #3: Yes

**********

3. Have the authors made all data underlying the findings in their manuscript fully available (please refer to the Data Availability Statement at the start of the manuscript PDF file)?

The PLOS Data policy

Reviewer #1: Yes

Reviewer #2: No

Reviewer #3: Yes

**********

4. Is the manuscript presented in an intelligible fashion and written in standard English?

Reviewer #1: Yes

Reviewer #2: Yes

Reviewer #3: Yes

**********

Reviewer #1: • LINE NUMBER 17: The short title “Screening Men in Toledo, Belize” is not comprehensive enough, as it doesn’t tell what type of screening was done in the study. I recommend other titles like “Hypertension and Diabetes Screening Among Toledo Men, Belize.”

• LINE NUMBERS 127–136: The study setting mainly described the facility where the data was going to be collected; however, it will be important to mention other important factors to consider, like the number of people that averagely visit the facility in from all the areas in the Toledo district and how easy it is for people to come from all of the areas in the district (road network and the centrality of the facility in relation to all the areas in the district).

• The analysis should include a clear relationship between blood pressure and blood glucose measurements, as world research shows a significant relationship between diabetes and hypertension.

• A suggestion on the variables measured in demographics of the study could have included the occupation, marital status, alcohol use, level of physical exercise, and living conditions. Also, another variable to be considered would be “the portion of participants that are already diagnosed with hypertension and/or diabetes and being managed.”

Reviewer #2: Many congratulations to the authors on the completion of the project and on putting out this report.

However, I have a few comments.

s/n Comments Recommendations

1. The introduction is largely well written, but has conflicting statements.

Line 85 suggests that disaggregated data are not available, hence the need for the study, but the paragraph starting from line 85 says otherwise. So one is unsure what the justification for the study really is. Review the introduction to clearly show the background, problem statement, and justification.

2. The objective of the study does not make it seem like research. An objective of merely screening men for certain conditions is too simplistic. Consider reviewing the objective to look less than just a medical outreach. What do you hope to achieve with the screening?

3. Methods are not clear on the sampling, sampling method, and sample size. It might be good to include all of these

4. Ethical Approval was unclear. If the approval from the university covers the area, there may not be a need to mention the one you did not get. Unless it does not, in which case you do not have ethical approval for this. Consider reviewing this.

In addition, the results and discussion would follow the objective (s), and in this case, it was unclear to follow. Based on this, the author could not have concluded as they did.

Reviewer #3: The candidate’s description of the problem under study is satisfactory. The candidate provided a good background to the study

The method section not clear. Need for more clarify in terms of the recruitment of participant

The conclusions address the objectives of the study.

**********

what does this mean?). If published, this will include your full peer review and any attached files.

**Do you want your identity to be public for this peer review?**  If you choose “no”, your identity will remain anonymous but your review may still be made public.

For information about this choice, including consent withdrawal, please see our Privacy Policy

Reviewer #1: No

Reviewer #2: No

Reviewer #3: No

**********

[NOTE: If reviewer comments were submitted as an attachment file, they will be attached to this email and accessible via the submission site. Please log into your account, locate the manuscript record, and check for the action link "View Attachments". If this link does not appear, there are no attachment files.]

Attachment Submitted filename: Comments for Manuscript Number PGPH-D-25-02214.docx

# Author comment: Author response to Decision Letter 1

**DOI:** 10.1371/journal.pgph.0007380.r002

3 Dec 2025

Attachment Submitted filename: Responses to Editor and Reviewers.docx

# Peer review: Decision Letter 1

**DOI:** 10.1371/journal.pgph.0007380.r003

**Authors:** Pengpeng Ye

10 Sep 2026

Screening Men in the Toledo District of Southern Belize for Elevated Blood Pressure and Blood Sugar

PGPH-D-25-02214R1

Dear Dr. Rovers,

We are pleased to inform you that your manuscript 'Screening Men in the Toledo District of Southern Belize for Elevated Blood Pressure and Blood Sugar' has been provisionally accepted for publication in PLOS Global Public Health.

Before your manuscript can be formally accepted you will need to complete some formatting changes, which you will receive in a follow up email. A member of our team will be in touch with a set of requests.

Please note that your manuscript will not be scheduled for publication until you have made the required changes, so a swift response is appreciated.

IMPORTANT: The editorial review process is now complete. PLOS will only permit corrections to spelling, formatting or significant scientific errors from this point onwards. Requests for major changes, or any which affect the scientific understanding of your work, will cause delays to the publication date of your manuscript.

If your institution or institutions have a press office, please notify them about your upcoming paper to help maximize its impact. If they'll be preparing press materials, please inform our press team as soon as possible -- no later than 48 hours after receiving the formal acceptance. Your manuscript will remain under strict press embargo until 2 pm Eastern Time on the date of publication. For more information, please contact globalpubhealth@plos.org.

Thank you again for supporting Open Access publishing; we are looking forward to publishing your work in PLOS Global Public Health.

Best regards,

Pengpeng Ye

Academic Editor

PLOS Global Public Health

***********************************************************

Reviewer Comments (if any, and for reference):

Reviewer's Responses to Questions

**Comments to the Author**

Reviewer #2: All comments have been addressed

**********

publication criteria?

Reviewer #2: Partly

**********

3. Has the statistical analysis been performed appropriately and rigorously?

Reviewer #2: I don't know

**********

4. Have the authors made all data underlying the findings in their manuscript fully available (please refer to the Data Availability Statement at the start of the manuscript PDF file)?

The PLOS Data policy

Reviewer #2: Yes

**********

5. Is the manuscript presented in an intelligible fashion and written in standard English?

Reviewer #2: Yes

**********

Reviewer #2: My condolences on the death of one of the authors.

**********

what does this mean?). If published, this will include your full peer review and any attached files.

**Do you want your identity to be public for this peer review?**  If you choose “no”, your identity will remain anonymous but your review may still be made public.

For information about this choice, including consent withdrawal, please see our Privacy Policy

Reviewer #2: No

**********
